# Re-Annotation Is an Essential Step in Systems Biology Modeling of Functional Genomics Data

**DOI:** 10.1371/journal.pone.0010642

**Published:** 2010-05-14

**Authors:** Bart H. J. van den Berg, Fiona M. McCarthy, Susan J. Lamont, Shane C. Burgess

**Affiliations:** 1 Department of Basic Sciences, College of Veterinary Medicine, Mississippi State University, Starkville, Mississippi, United States of America; 2 Institute for Digital Biology, Mississippi State University, Starkville, Mississippi, United States of America; 3 Life Sciences and Biotechnology Institute, Mississippi Agriculture and Forestry Experiment Station, Starkville, Mississippi, United States of America; 4 The AgBase Databases, Mississippi State University, Starkville, Mississippi, United States of America; 5 Department of Animal Science and Center for Integrated Animal Genomics, Iowa State University, Ames, Iowa, United States of America; Miami University, United States of America

## Abstract

One motivation of systems biology research is to understand gene functions and interactions from functional genomics data such as that derived from microarrays. Up-to-date structural and functional annotations of genes are an essential foundation of systems biology modeling. We propose that the first essential step in any systems biology modeling of functional genomics data, especially for species with recently sequenced genomes, is gene structural and functional re-annotation. To demonstrate the impact of such re-annotation, we structurally and functionally re-annotated a microarray developed, and previously used, as a tool for disease research. We quantified the impact of this re-annotation on the array based on the total numbers of structural- and functional-annotations, the Gene Annotation Quality (GAQ) score, and canonical pathway coverage. We next quantified the impact of re-annotation on systems biology modeling using a previously published experiment that used this microarray. We show that re-annotation improves the quantity and quality of structural- and functional-annotations, allows a more comprehensive Gene Ontology based modeling, and improves pathway coverage for both the whole array and a differentially expressed mRNA subset. Our results also demonstrate that re-annotation can result in a different knowledge outcome derived from previous published research findings. We propose that, because of this, re-annotation should be considered to be an essential first step for deriving value from functional genomics data.

## Introduction

Integrating and modeling ‘omics’ datasets in systems biology facilitates biological understanding at a molecular systems level. Biological systems are studied from global gene, transcript, protein, protein interaction and metabolite levels. Microarray technology advanced functional genomics by facilitating high-throughput acquisition of large functional genomics datasets [1], [2], [3]. We can derive a system-level understanding from functional genomics data through modeling based, for example, on Gene Ontology (GO) [4] as well as canonical pathway and network analyses.

Up-to-date gene product structural- and functional- annotations (i.e. identifying the genes represented on microarrays and linking these to functional information, respectively) are an essential foundation of systems biology modeling. The primary repository for structural annotations of most commercial and custom-made microarrays, and their related studies, is the National Center for Biotechnology Information (NCBI) Gene Expression Omnibus [5]. Structural annotations are assigned during microarray development and initial publication. However, these structural annotations are not always regularly updated after publication. With the acquisition of new genomics data and development of annotation tools, the structural- and functional-annotation databases are updated on a regular basis [6], [7], [8].

For species for which genomic sequences are newly available, the rapidity of updates of annotation data and the increase in high-throughput experimental platforms, such as microarrays, is astounding. The challenge of appropriately managing and, especially, interpreting experiment-based datasets will be especially difficult for those species with small research communities, such as ecologically important or agricultural animal species. The number of animal species completely sequenced and published before 2003 was 6 (*Drosophila melanogaster*, *Anopheles gambiae*, *Mus musculus*, *Homo sapiens*, *Caenorhabditis elegans*, and *Caenorhabditis briggsae*). From 2003 to present, 103 animal genomes are completely sequenced and published, and this trajectory of data generation is expected to increase. Chicken is one animal that exemplifies the rapidly evolving structural and functional annotations. The chicken UniGene clustering database, the major repository for structural annotation of ESTs, was first released in 2003, and was developed up to build 40 used here. This is an average of one update every two months. The GO consortium is the primary repository for functional annotations. GO functional annotations are continually updated and on average there is a new chicken GOA database released monthly; from June 5^th^ 2004 there have been 46 releases.

Disregarding this new and/or corrected information will limit the power of using functional genomics methods and hinder comprehensive system-level modeling. It is logical to first update the structural and functional annotations of functional genomics datasets before they are used as part of a systems biology modeling paradigm. Although genome-wide gene structural re-annotation has proven valuable [9], [10], [11], [12], [13], [14], [15], [16], [17], [18], a corresponding genome-wide gene functional re-annotation is not frequently considered.

Here we use the FHCRC Chicken 13K cDNA v.2.0 microarray (GPL 1836) [19] as an example of both the power gain and necessity of updating genome annotations for accurate modeling. This array has been used to characterize global gene expression for cancer [20] [21] [22], host-pathogen interactions [23] [24], and developmental biology [25]. The FHCRC microarray was developed and structurally-annotated in 2004. After publication in February 2005, the structural annotations were updated only once in January 2006 (GEO accession GPL2863). However, since the release of the chicken genome in 2004 [26], new and/or corrected structural- and functional- annotations have been assigned [27] [28] [29] [30] [31] [32]. We re-annotated the FHCRC chicken 13K cDNA v2.0 microarray and reanalysed a previously published differentially expressed mRNA experimental dataset generated with this microarray [24]. We compared the quality of the new annotations with that of the prior annotations as well as the results of modeling. We show that re-annotation not only provides more structural- and functional-annotations but also improves the power of functional genomics modeling, and that re-analysis after re-annotation can provide different interpretations compared with data whose annotation is less optimal.

## Results

All structural mappings, GO term assignments and pathway analysis are available on the AgBase website at http://agbase.msstate.edu/tools/reannotation/.

### Structural re-annotation

Table 1 shows the results for the structural re-annotation of the whole FHCRC chicken 13K cDNA v2.0 (GPL 1836) microarray and the differentially expressed mRNA data from Zhou et al. [24]. For the whole microarray, 15,609 ESTs were listed originally in the microarray data table. Because the ESTs are generated from chicken cells and tissues, we aimed to retrieve only chicken structural annotations. We were able to increase the chicken-specific annotations (i.e. annotate an EST to a chicken gene) by 9.8-fold. Even though structural annotations could not be assigned for all ESTs in the re-annotated dataset, the number of ESTs with no structural annotations was reduced to 15% of the original ESTs with no structural annotation. The total number of unique chicken genes assigned to the whole array was improved by 10.5-fold.

**Table 1:** Structural re-annotation results.

	Whole microarray data			Differentially expressed mRNA data		
Structural annotation	Original	Re-annotated	Fold Δ	Original	Re-annotated	Fold Δ
Total EST	15,609	15,227	0.98	57	54	0.95
EST to Chicken gene	1,457	14,206	9.75	16	49	3.06
EST to Human gene	3,951	0	n/a	13	0	n/a
EST to Mouse gene	1,487	0	n/a	5	0	n/a
EST to Rat gene	450	0	n/a	3	0	n/a
EST to genes other species	1,409	0	n/a	7	0	n/a
EST with no gene annotation	6,855	1,032	0.15	13	5	0.38
Total unique chicken gene annotations	1,136	11,868	10.45	16	43	2.69

The results of structural annotation are compared between the original annotation data and the re-annotation dataset for both the whole microarray as for the differentially expressed mRNA dataset. Re-annotation increased EST to chicken gene mapping and total unique chicken genes, in both the whole microarray as for the differentially expressed mRNA dataset, while reducing need for structural annotations based on orthology.

For the differentially expressed mRNAs, 57 were originally identified to play a significant role in the host-pathogen response within a *Salmonella enterica* Serovar *enteritidis*-challenged chicken model [24]. We retrieved 54 unique identifiers from these ESTs and re-annotated them with *ArrayIDer*. We mapped 49 ESTs to corresponding chicken genes, which is an increase of 3.1-fold compared to the original data. Only 5 of the 54 ESTs did not have structural annotations, compared to 13 in the original dataset. The total unique chicken gene annotations were increased by almost 2.7-fold.

### Functional re-annotation

Table 2 shows the results for the functional re-annotation of the whole FHCRC 13K chicken cDNA microarray and the differentially expressed mRNA data from Zhou et al. [24]. We retrieved all possible proteins for the total number of genes annotated on the whole microarray. We used the GOA chicken database build 17 as baseline for functional annotations available at the time of the study reported by Zhou et al. [24]. We used the GOA chicken database build 46 as a functional annotation resource for the re-annotation. Originally, 785 unique proteins were identified of which 615 proteins had functional annotations assigned in the GOA chicken database build 17. We retrieved 15.1-fold more chicken proteins for the whole microarray after re-annotation and the total number of proteins that had functional annotations assigned was increased by more than 6.2-fold.

**Table 2:** Functional re-annotation results.

	Whole microarray data			Differentially expressed mRNA data		
Functional annotation	Original	Re-annotated	Fold Δ	Original	Re-annotated	Fold Δ
Total unique chicken protein annotations	785	11,868	15.10	12	43	3.58
Total proteins GO annotated	615	3,845	6.25	9	38	4.22
Total GO terms	3,929	27,815 (9,595)	7.08 (2.44)	39	365 (190)	9.36 (4.87)
Unique GO terms	1,050	2,652 (1,662)	2.53 (1.58)	26	160 (92)	6.15 (3.54)
Total GAQ score	43,245	305,996 (107,006)	7.08 (2.47)	375	4,158 (2663)	11.08 (6.04)
Mean GAQ score	70	80 (174)	1.13 (2.49)	42	109 (296)	2.63 (7.05)
GO depth score	21,142	143,206 (51,391)	6.77 (2.43)	177	1,921 (934)	10.85 (5.28)
∑GO annotation confidence score including IEA	8,037	57,696 (20,040)	7.18 (2.49)	81	781 (527)	9.64 (6.51)
∑GO annotation confidence score excluding IEA	1,325	14,258 (3,460)	10.76 (2.61)	5	143 (283)	28.60 (56.6)

The results of functional annotation are compared between the original annotation data and the re-annotation dataset for both the whole microarray as for the differentially expressed mRNA dataset. In addition, re-annotation increased the number of GO terms, the total GAQ score, the detail and the confidence in the GO annotations assigned. The numbers in parentheses represent the re-annotation results of only the original 615 chicken proteins of the whole array data. This score represents the standard baseline of the impact of re-annotation improvement. For the differentially expressed mRNA data, the numbers in parentheses represent the re-annotation results of the original 9 differentially expressed mRNAs.

The re-annotation not only increased the number of structural annotations, but also greatly increased the number of functional annotations. The total number of GO terms represented by the retrieved proteins increased more than 7.0-fold and the total number of unique GO terms by more than 2.5-fold. To quantify the quality of the functional annotations assigned to our re-annotated data set we calculated the GAQ score. The GAQ score consists of the total number of proteins, the GO term depth in the GO tree and the assigned evidence code for the GO annotation [33]. The total GAQ score for the retrieved GO annotations improved more than 7.0-fold (P<0.235).

Although we greatly increased the number of GO annotations, the increase of total GAQ score from 43,245 to 305,996 is not statistically significantly different because of the large number of GO annotations assigned with the lower scoring evidence codes ‘ND’ (No Data) and ‘IEA’ (Inferred by Electronic Annotation). However, the mean GAQ score (i.e. total GAQ score/total number of unique proteins) was statistically significantly increased by 13% compared to the original (P<0.002) i.e. the GO annotation quality per protein improved after re-annotation. The GO depth score improved more than 6.5-fold, demonstrating an increased level of biological detail for the re-annotated dataset. The overall GO annotation confidence score, (a measure of the difference in quality of different types of annotations) improved more than 7.1-fold. In addition, to assess the confidence improvement without the down-weighting caused by IEA evidence code scores in the total GAQ score, we calculated the GO annotation confidence score excluding IEA evidence code scores. The GO annotation confidence score based on annotations that themselves are based on data derived from experimental assays improved more than 10.7-fold, demonstrating that we can be more confident about the assigned annotations in the re-annotated whole microarray dataset.

The impact of re-annotation is especially well demonstrated for the 615 proteins that were originally annotated to chicken (numbers in parentheses in Table 2). Even though these proteins were the best-annotated previously, we improved the total number of GO terms almost 2.5-fold and the number of unique GO terms by almost 60%. The total GAQ and mean GAQ scores both increased by almost 2.5-fold (P<0.044 and P<2.7e-78, respectively). This results in greater depth and confidence in the knowledge represented by these 615 proteins as demonstrated by the almost 2.5-fold increase of in total GO depth and total GO annotation confidence score. The GO annotation confidence score calculated excluding IEA evidence code annotations improved over 2.6-fold.

For the differentially expressed mRNA data, we retrieved almost 3.6-fold more proteins, with 9.4-fold more total GO terms, and over 6.1-fold more unique GO terms. Similar to the whole array, we calculated the total and mean GAQ scores for the differentially expressed mRNA dataset and this increased more than 11.0- and 2.6-fold respectively (P<0.023 and P<5.4e-6, respectively). The total GO depth score and total GO annotation confidence score both increased by more than 10.8- and 9.6-fold respectively.

### GOSlim modeling

**Figure 1:**
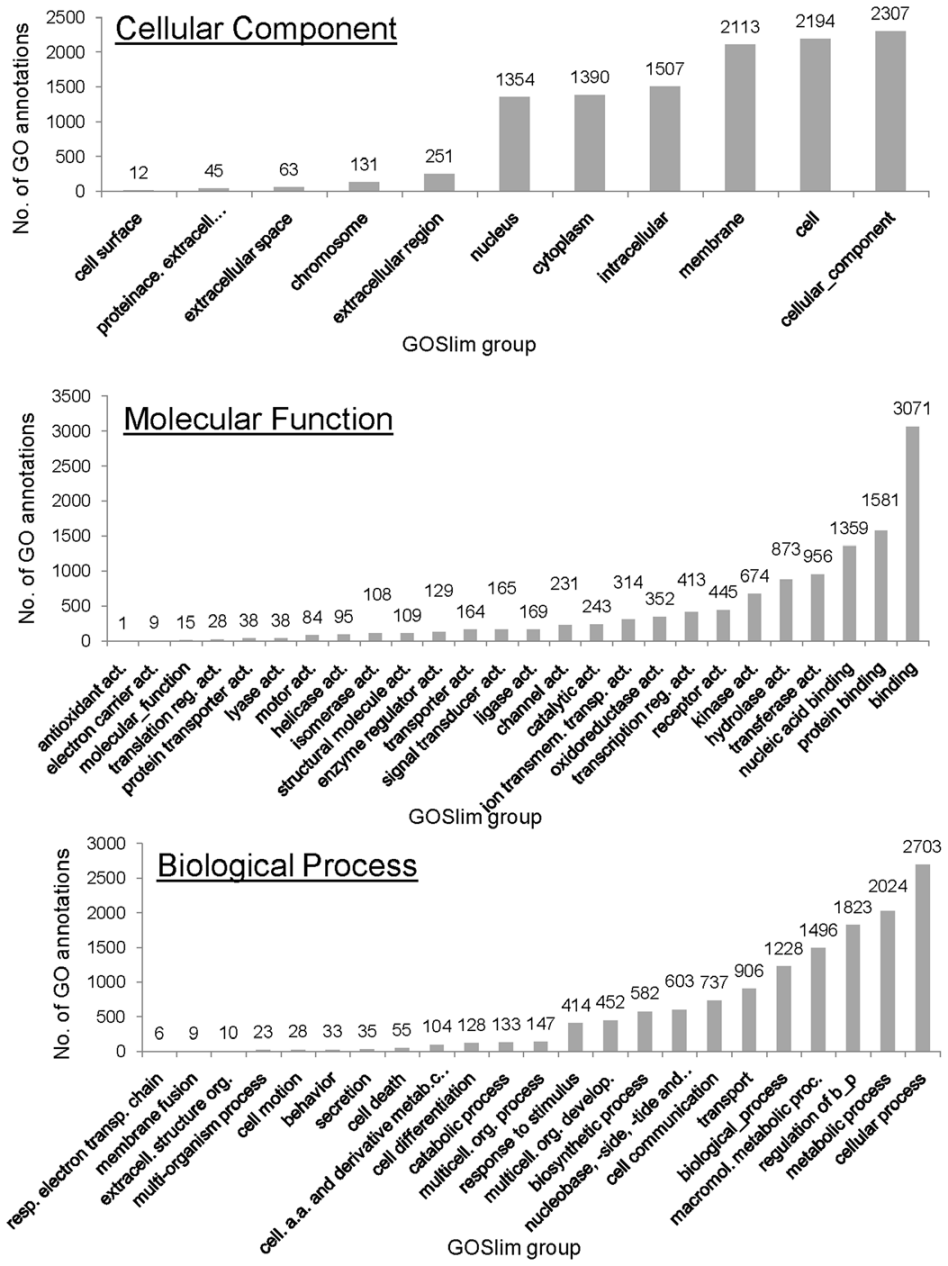
Whole microarray GOSlim modelling. The difference in number of GO annotations in the GOSlim groups for the GO ontologies ‘cellular component’, ‘molecular function’ and ‘biological process’ between the original and re-annotated whole microarray gene dataset. The whole microarray GOSlim modeling shows that re-annotation increases the number of GO annotations in each GOSlim group for each ontology.

**Figure 2:**
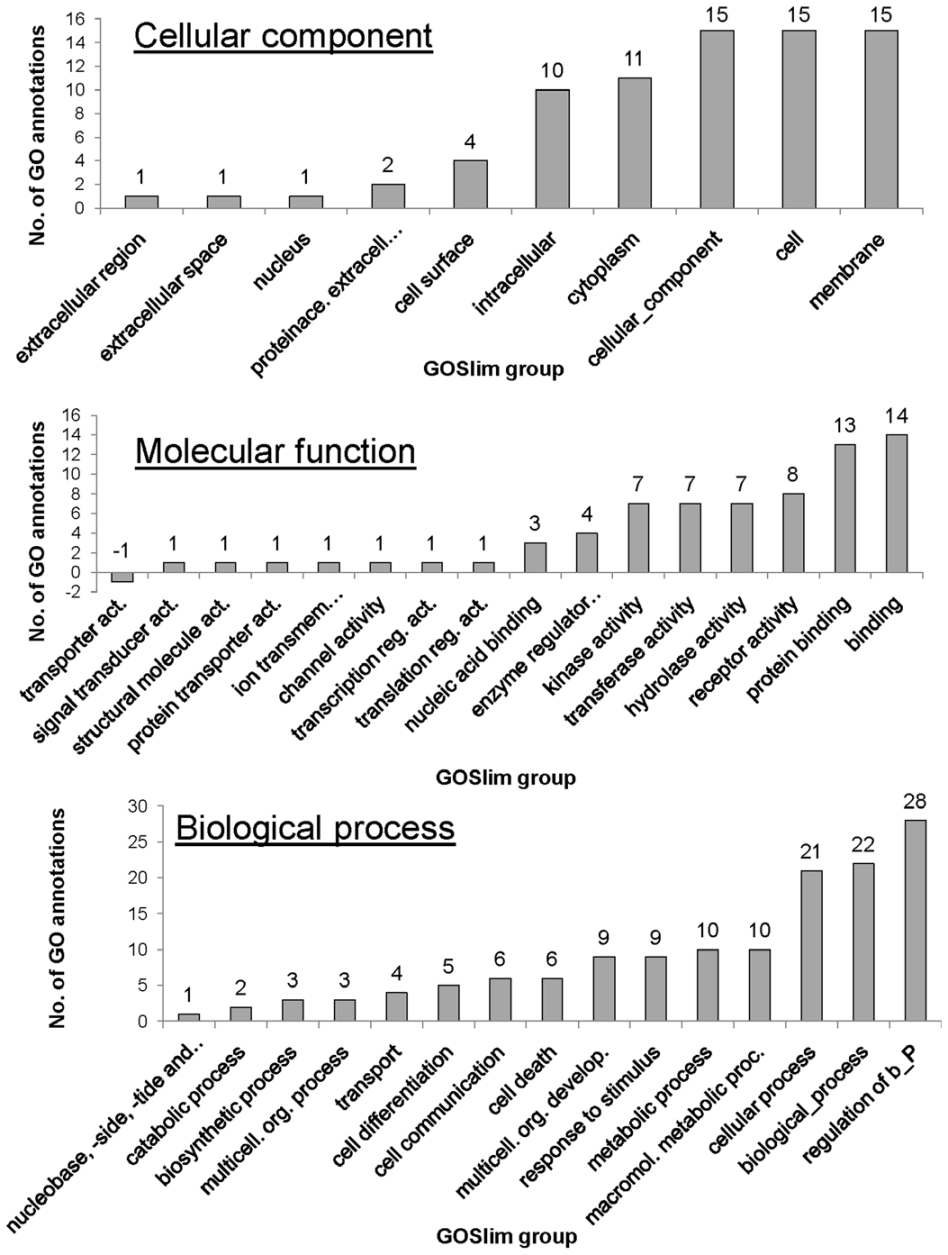
Differentially Expressed mRNA GOSlim modelling. The difference in number of GO annotations in the GOSlim groups for the GO ontologies ‘cellular component’, ‘molecular function’ and ‘biological process’ between the original and re-annotated differentially expressed mRNA dataset. The differentially expressed mRNA GOSlim modeling shows that re-annotation increases the number of GO annotations for most GOSlim group. The negative value for the GOSlim group ‘transporter activity’ in the ‘molecular function’ ontology are caused by updated GO annotations to the more detailed ‘protein transporter activity’ GOSlim group.

We generated GOSlim models for cellular component [CC], molecular function [MF] and biological process [BP] Gene Ontology for the microarray and differentially expressed mRNA dataset to visualize the major functional groups represented. We used the “GOA and whole proteome” GOSlim set [34]. Figure 1 shows the net difference between the re-annotated and original GOSlim distribution for the whole microarray. After re-annotation, all CC, MF and BP GOSlim groups contain more GO annotations. The GOSlim groups “cellular_component”, “molecular_ function” and “biological_process” also include GO annotations made to the GO term “component unknown”. Figure 2 shows the difference between the re-annotated and original GOSlim distribution for the differentially expressed mRNA dataset. After re-annotation, all CC GOSlim groups contain more GO annotations; 15 out of 16 MF GOSlim groups contain more GO annotations; and all BP GOSlim groups contained more GO annotations. The negative value for the GOSlim group “transporter activity” is due to the re-distribution of the GO annotation to the more detailed GOSlim group “proteins transporter activity”. Such re-distribution occurs when new GO annotation becomes available or when older GO annotations are updated. For example, chicken CD3 epsilon (UniProtKB accession Q98910) was integrated to the UniProtKB/Swiss-Prot database on the November 1, 1997, yet received its first GO annotation on the November 4, 2008 and the second GO annotation on November 25, 2008. Regardless, the large differences before and after re-annotation show that re-annotation shifted the balance of genes represented on the array from developmental to metabolic processes.

### Pathway and molecular network analysis

We used Ingenuity Pathway Analysis (IPA) to retrieve and compare the significant genetic pathways and networks able to be modeled by both the original and re-annotated whole microarray and differentially-expressed mRNA datasets. We identified 133 pathways common to both the original and re-annotated whole microarray dataset. Although these pathways are all shared, the pathway coverage was increased 6.9-fold, with a coverage variance of only 49% of the original variance. We identified 35 pathways unique to the original dataset and 37 unique to the re-annotated data. However, unique original dataset pathways were identified with 91 genes and mean coverage of 4.2%. In contrast, unique re-annotated dataset pathways were identified with 608 genes and mean coverage of 25.4%. Table 3 and 4 lists the top 10 significant pathways identified in the original (Table 3) and re-annotated (Table 4) whole array datasets. Only two out of ten pathways are shared (indicated in bold). The mean pathway coverage ratio for the pathways (i.e. number of genes in datasets/total number of genes in pathway) represented for the original annotations is 0.090 but for the re-annotated data is 0.445 (i.e. 5-fold increase).

**Table 3:** Top 10 significant pathways original whole microarray dataset.

Rank	Pathway Original	Ratio coverage	# Genes from dataset
1	**Apoptosis Signaling**	1.17E-01	11
2	Axonal Guidance Signaling	6.42E-02	26
3	**Integrin Signaling**	8.37E-02	17
4	Amyotrophic Lateral Sclerosis Signaling	9.09E-02	10
5	Actin Cytoskeleton Signaling	7.14E-02	17
6	CDK5 Signaling	9.68E-02	9
7	Caveolar-mediated Endocytosis	9.76E-02	8
8	Neurotrophin/TRK Signaling	1.15E-01	9
9	VEGF Signaling	9.28E-02	9
10	Clathrin-mediated Endocytosis	7.19E-02	12
**MEAN**		**9.01E-02**	

Top 10 of significant pathways identified by Ingenuity Pathway Analysis for the original whole microarray dataset. Pathways in bold were found in the top 10 series of both the original and re-annotated datasets. Ratio Coverage  =  the number of genes from the data set that map to the pathway divided by the total number of genes that map to the canonical pathway is displayed (Ingenuity® Systems).

**Table 4 pone-0010642-t004:** Top 10 significant pathways re-annotated whole microarray dataset.

Rank	Pathway Original	Ratio coverage	# Genes from dataset
1	CD28 Signaling in T Helper Cells	4.52E-01	56
2	**Integrin Signaling**	4.68E-01	95
3	NF-κB Signaling	4.29E-01	63
4	Insulin Receptor Signaling	4.49E-01	62
5	**Apoptosis Signaling**	4.79E-01	45
6	IL-9 Signaling	4.59E-01	17
7	Role of NFAT in Regulation of the Immune Response	3.72E-01	70
8	Angiopoietin Signaling	4.17E-01	30
9	Ceramide Signaling	4.76E-01	40
10	Virus Entry via Endocytic Pathways	4.48E-01	43
**MEAN**		**4.45E-01**	

Top 10 of significant pathways identified by Ingenuity Pathway Analysis for the re-annotated whole microarray dataset. Pathways in bold were found in the top 10 series of both the original and re-annotated datasets. Ratio Coverage  =  the number of genes from the data set that map to the pathway divided by the total number of genes that map to the canonical pathway is displayed (Ingenuity® Systems).

We identified 34 pathways shared between the original and re-annotated differentially expressed mRNA datasets. Similar to the whole array, the pathway coverage in the re-annotated dataset was increased 6.3-fold, with a coverage variance of 61% of the original variance. Fourteen pathways were unique to the re-annotated differentially expressed mRNA dataset. Table 5 and 6 lists the top 10 of the significant shared pathways identified in the original (Table 5) and re-annotated (Table 6) differentially expressed mRNA datasets. The mean pathway coverage ratio for the pathways (i.e. number of genes in datasets/total number of genes in pathway) represented for the original annotations is 0.018 but for the re-annotated data is 0.030 (i.e. 1.6-fold increased). Re-annotation improves canonical pathway coverage and shifts the pathway identification significance. Three out of the top ten pathways are shared (Table 5 and 6, bold) between the original and re-annotated datasets.

**Table 5 pone-0010642-t005:** Top 10 significant pathways original differentially expressed mRNA dataset.

Rank	Pathway Original	Ratio coverage	Genes from dataset
1	**Hepatic Fibrosis/Hepatic Stellate Cell Activation**	2.22E-02	CCL5, FN1, IL1B
2	Acute Phase Response Signaling	1.12E-02	FN1, IL1B
3	**Glucocorticoid Receptor Signaling**	1.08E-02	CD3E, CCL5, IL1B
4	**Role of Cytokines in Mediating Communication between Immune Cells**	1.79E-02	IL1B
5	Cytotoxic T Lymphocyte-mediated Apoptosis of Target Cells	3.7E-02	CD3E
6	Docosahexaenoic Acid (DHA) Signaling	2.22E-02	IL1B
7	TREM1 Signaling	1.45E-02	IL1B
8	IL-10 Signaling	1.41E-02	IL1B
9	Calcium-induced T Lymphocyte Apoptosis	1.61E-02	CD3E
10	LXR/RXR Activation	1.18E-02	IL1B
**MEAN**		**1.78E-02**	

Top 10 of significant pathways identified by Ingenuity Pathway Analysis for the original differentially expressed mRNA dataset. Pathways in bold were found in the top 10 series of both the original and re-annotated datasets. Genes in bold and italics were only identified in the re-annotated dataset. Ratio Coverage  =  the number of genes from the data set that map to the pathway divided by the total number of genes that map to the canonical pathway is displayed (Ingenuity® Systems).

**Table 6:** Top 10 significant pathways re-annotated differentially expressed mRNA dataset.

Rank	Pathway Original	Ratio coverage	Genes from dataset
1	Cytotoxic T Lymphocyte-mediated Apoptosis of Target Cells	7.41E-02	CD3E, ***FAS***
2	**Hepatic Fibrosis/Hepatic Stellate Cell Activation**	3.7E-02	CCL5, FN1, IL1B, ***FAS***, ***LY96***
3	CCR5 Signaling in Macrophages	3.45E-02	CD3E, CCL5, ***FAS***
4	Death Receptor Signaling	3.08E-02	BIRC2
5	Induction of Apoptosis by HIV1	3.03E-02	BIRC2, ***FAS***
6	**Glucocorticoid Receptor Signaling**	1.44E-02	CD3E, CCL5, IL1B, ***ANXA1***
7	p38 MAPK Signaling	2.11E-02	IL1B, ***FAS***
8	CTLA4 Signaling in Cytotoxic T Lymphocytes	2.25E-02	CD3E, ***AP1G1***
9	Apoptosis Signaling	2.13E-02	BIRC2, ***FAS***
10	**Role of Cytokines in Mediating Communication between Immune Cells**	1.79E-02	IL1B
**MEAN**		**3.04E-02**	

Top 10 of significant pathways identified by Ingenuity Pathway Analysis for the re-annotated differentially expressed mRNA dataset. Pathways in bold were found in the top 10 series of both the original and re-annotated datasets. Genes in bold and italics were only identified in the re-annotated dataset. Ratio Coverage  =  the number of genes from the data set that map to the pathway divided by the total number of genes that map to the canonical pathway is displayed (Ingenuity® Systems).

Intuitively the re-annotated set should contain pathways not detected in the original analysis; however, almost as many pathways were unique to the original analysis by Zhou et. al. [24] as were unique to analysis of the re-annotated data set. This is a property of the statistical methods used by IPA. In short, IPA identifies pathways using a Fisher's exact test to calculate the probability that the association between the genes in the dataset and the canonical pathway is explained by chance alone relative to all other pathways in the database chosen for interrogation (Ingenuity® Systems, www.ingenuity.com), but not relative to the proportional coverage of the pathway. The proportion of proteins in a given pathway is given by the “ratio coverage” i.e. the number of genes from the data set that map to the pathway divided by the total number of genes that map to the canonical pathway. Re-annotation resulted in 4.5- and 1.7-fold improvements in mean pathway ratio coverage in the entire and differentially-expressed datasets, respectively (Tables 3 through [Table pone-0010642-t004] [Table pone-0010642-t005] 6) and thus greater confidence in the pathways identified after re-annotation.

## Discussion

Comprehensive and accurate structural and functional annotation is fundamental for modeling functional genomics data to derive biological knowledge. Commercial and custom-spotted microarrays are often annotated before their publication and are sometimes not updated to provide the most recent and corrected structural and functional annotations. We demonstrate that re-annotation of a microarray provided not only more information but also better statistical confidence in the functional annotations.

An important distinction is that between genome re-annotation and data set re-annotation. Most references use the term “re-annotation” in the context of genome re-annotation (i.e. updating annotations in a genomic database). The assumption being that the genome annotation is current. In contrast, here we have re-annotated a functional genomics data set itself. However, we are aware that a potential issue confounding functional genomics data re-annotation is annotation error in the genomic databases [35] [36] [37] [38].

Although structural and functional re-annotation of functional genomic datasets should be intuitive we have not seen that doing such a re-annotation is commonly reported in the material and methods section of the published literature. Here we aimed to provide a quantitative example of the importance of such re-annotation (especially when working in a less widely-used model species). Previously this same dataset was structurally re-annotated when we designed an automated method (*ArrayIDer*) for microarray [32] structural re-annotation. But because of changes in annotation over the less than 12 months since this annotation, we used *ArrayIDer* to further update these structural annotations. In the original publication, only 1131 ESTs were structurally annotated based on chicken genes, while the remainder of the microarray was structurally annotated using orthologs from 249 different species [19]. We not only retrieved more structural annotations, but also improved the specificity of these structural annotations by assigning structural annotations from chicken only. In addition to updating the structural annotations, we also did manual curation of poorly annotated ESTs to improve the structural annotation breadth. The ESTs without any current structural annotations are candidates for orthology-based structural annotation. We did not attempt to retrieve any orthologs for the annotated ESTs as this adds the variable of ortholog assignment [39] [40] [41], which was beyond the scope of this work.

While it may be interesting to compare our GAQ score-based quantitative analysis with another metric for measuring for functional annotation quality, no similar method exists. The Genome Annotation Score (GAS) [42], the Gene Characterization Index (GCI) [43] and the GeneCard Inferred Functionality Score (GIFtS) [44] are additional algorithms that assess gene annotation quality. The GAS algorithm weighs the annotation evidence code in its GO annotation scoring, but it only distinguishes automatically- from manually-assigned annotations. In comparison, the GAQ weighs each annotation evidence code separately and the automatically assigned evidence code is lowest scoring. In addition, GAS only calculates an average, whole genome quality score, while GAQ calculates the quality score for each gene, so providing higher resolution for the annotation quality scoring. Similarly, the GCI algorithm does not take the annotation evidence code into consideration.

The GIFtS algorithm calculates a gene's annotation quality score using a binary vector system (either ‘0’ or ‘1’) representing either the presence or absence of data. In effect, if one gene has 3 annotations and another gene 10 annotations, both will be scored equally. In contrast, the GAQ scoring algorithm assigns scores for each annotation for a particular gene individually and so better annotated genes score higher.

In addition, GAQ uses the ‘depth’ of a GO term in the GO acyclic graph as a quantitative measure for the level of annotation detail, the GAS, GCI and GIFtS algorithms do not take the GO annotation's level of detail in consideration. Finally, unlike the GAQ algorithm, GAS does not allow direct input of large numbers of gene product accession numbers and, although GIFtS and GCI can do so, both algorithms are limited only to human genes and requires ortholog-searching to be used for any other species.

We used the GAQ score to assess the improvement in functional annotations after re-annotation of the FHCRC Chicken 13K cDNA v2.0 microarray and a differentially expressed mRNA set from a previous study using this microarray. At the time of writing, 97.1% of all chicken GO annotations in the GOA database are “Inferred by Electronic Annotation” (IEA). Because we have more functional annotations (‘breadth’) in the re-annotated whole FHCRC microarray dataset, the proportion of IEA, together with the higher number of proteins in the re-annotated dataset, cause the 7.08-fold increase of the total GAQ score not to be significant. However, even though the mean GAQ score increased only by 13%, this increase of the mean GAQ score is a marked improvement compared to the original annotation.

In addition, the GAQ score down-weights annotations inferred electronically compared to those inferred by experimental assays. Were we to exclude the IEA annotations then the power to model the data would reduce because fewer proteins would have annotations (i.e. annotation “breadth” decreases); but the mean GAQ for each gene would increase because the lower scoring proteins would be excluded. Regardless, IEA is a valid method to annotate to the GO and, so long as the evidence code is kept in mind during modeling, then we consider IEA annotations valuable additions—especially in model organisms other than mouse. For this reason we included the GO annotation confidence score (a measure of the difference in quality of different types of annotations) calculated without the lowest scoring, most common IEA annotations, and doing so demonstrates that re-annotation even without IEA is an improvement.

We calculated the GO annotation confidence score excluding IEA evidence code scores to measure the improved annotation without the down-weighting caused by IEA evidence code scores (Table 2).

For the original whole microarray annotation, the ratio of the GO annotation confidence score including and excluding IEA, before re-annotation is 6.1 (8037 vs. 1325) and after re-annotation 5.8 (20040 vs. 3460). For the differentially expressed mRNA annotation, the ratio of the GO annotation confidence score including and excluding IEA, before re-annotation is 9.1 (81 vs. 5) and after re-annotation 1.9 (527 vs. 283).

Although the general trend is similar before and after re-annotation of the original whole microarray dataset, there is an exception for the differentially expressed mRNA dataset. We believe that this difference is due to the structural re-annotation of mRNA clones to corrected, up-to-date structural annotations with less functional annotations assigned.

Originally, clone pat.pk0035.g9.f was structurally annotated as Ribonuclease homolog precursor (RSFR; Gga.46257) and functionally annotated in chicken GOA database build 17 with only 4 annotations were assigned, all based on IEA evidence. When this gene is re-annotated using chicken GOA database build 46, 66 annotations were assigned, of which only 12 were IEA-based. The total GO annotation confidence score for this particular gene is 226, representing 80% of the total GO annotation confidence score (283) of all 9 genes functionally re-annotated to chicken GOA database build 46. Re-annotation structurally annotates clone pat.pk0035.g9.f as “Marker protein” (Ch21; Gga.739). This gene is functionally annotated in chicken GOA database 46 to 22 annotations, all based on IEA evidence.

GOSlim sets are designed to summarize GO datasets and, although this approach loses detailed functional information, it is suitable for comparing and visualizing the overall effect of functional re-annotation. The whole-microarray GOSlim modeling showed increases in GO annotation for all GOSlim groups for each of the three GO ontologies. However, re-annotation results in more unique GO annotations and increased GO depth, thus more GO annotation detail. Summarizing these GO annotations to relevant, more global GOSlim groups results in a higher GO annotation count for global GO terms for GO Cellular Component ontology terms such as “cellular_component” or “cell”. Similarly for GO Molecular Function, this phenomenon is shown for the GO group “binding”, which is a global GO term that accounts for 74.4% of the chicken gene products in the Molecular Function ontology.

GOSlim modeling of the differentially expressed mRNA dataset showed both increased and decreased GOSlim groups. In some instances this reduction is a direct result of re-annotation. For example, the number of GO annotations summarized to ‘transporter activity’ decreases, but in contrast the more specific GO terms ‘protein transporter activity’ and ‘ion transmembrane transporter activity’ have more GO annotations. As expected, re-annotation results in increased GO annotation granularity or specificity for the differentially expressed mRNA dataset.

Because the higher order GO terms used in the GOSlim analysis do not describe in detail which canonical pathways and networks are represented by the datasets, we used Ingenuity Pathway Analysis (IPA) to retrieve all significant canonical pathways. We clearly increased the canonical pathway coverage and lowered statistical variance in assigning pathways.

In order to calculate the statistical variance using the Fisher's exact test, we used the Agilent 44K chicken microarray as reference list, which is closest to the FHCRC 13k chicken cDNA microarray. Although not optimal, this approach provides the best evaluative means for assessing the impact of re-annotation on functional genomics data. In addition, IPA uses the Fisher's exact test for significance calculations, which assumes gene-independence. However, in biology, gene expression cannot always, or arguably is never, independent [45]. Regardless, the method we used does provide a standard system for comparing pre and post re-annotation and there is a clear difference.

At the time of manuscript preparation, the most current publicly available structural and functional annotations were used. We improved the total structural annotations by 10.5 fold, the functional annotations by about 6.3 fold and the pathway coverage by 6.9 fold, since the last update of the FHCRC array in 2006. The time period from the update until our data analysis covers 40 months, which represents 20 UniGene updates, 40 GOA chicken database updates and 13 IPA database updates. This continual updating suggests that re-annotation of our annotations would again be necessary in about 4 to 6 months.

Even though it is clear that re-annotation has a significant effect on data quality, the most important question for knowledge generation is whether or not it has an impact on data interpretation. The FHCRC 13K chicken cDNA microarray has been used for cancer [21] [22], growth and development [25], and host-pathogen interaction [23] [24] research. We re-annotated differentially expressed mRNAs identified in work using the FHCRC 13K chicken cDNA microarray to study genetic differences in chicken responses to a *Salmonella enterica* Serovar *enteritidis* infection [24]. This work reported several candidate genes for genetic resistance to *Salmonella* infection. The impact of our re-annotation on interpretation of this study can be described on three levels.

First, the previous annotation allowed only for candidate gene identification and, as stated by Zhou et al., in the paper, one constraint at the time was lack of annotation allowing in-depth analysis of signal transduction pathways. Our re-annotation increased the pathway coverage of several major immune response pathways (Table 6), which allows more comprehensive modeling of signalling pathways. One example is the re-annotation of the FAS molecule. Originally, Zhou et al. did not have any nucleotide accession numbers assigned for the FAS gene, which hindered retrieval of additional cross-reference gene information from public databases and down-stream pathway modeling. Our re-annotation provides the necessary cross-reference information and allows identification of canonical pathways involving FAS (Table 5 and 6).

Second, the re-annotated data allows us to confirm and consolidate suggestions from Zhou et al. For example, differential expression of CD3epsilon, cytokine IL-1β, and chemokines ah294 (CCL5) were identified as key genes involved in the immune response to SE. The re-annotated data not only allowed chicken-specific functional annotation of these genes, but allowed identification of their related pathways with greater confidence and coverage (Table 5 and 6).

Third, re-annotation identified additional genes involved in major immune pathways that were not identified in the original work. For example, Zhou et al., identified differential regulation of two ESTs (pat.pk0028.f8.f and pat.pk0032.e7.f), which both were structurally annotated to the T-cell surface glycoprotein CD28. Re-annotation, however, showed that the EST pat.pk0028.f8.f (GenBank AI980641) is more correctly structurally annotated to protein tyrosine phosphatase type IVA member 1 (PTP4A1) and EST pat.pk0032.e7.f (GenBank AI980751) is more correctly structurally re-annotated to inducible T-cell co-stimulator (ICOS or CD287). ICOS is interesting in that it shares structural and functional similarities with CD28 and both are required for naïve CD4+ T-cell activation, yet ICOS contributes more to T cell survival and proliferation during an immune response [46] [47] [48]. In addition, ICOS stimulates IL-10 expression, which in turn influences B-cell immunity [47] [49]. The EST that we structurally re-annotated to ICOS, had increased expression in chickens susceptible to SE infection with high SE burden. Increased ICOS expression is linked to immune deregulation in several human diseases including those of the gastrointestinal tract [47], in murine colitis [50] and specifically, in *Salmonella enterica* serovar *Typhimurium* infection in mice [51]. With the updated annotation, therefore, we can now, demonstrate something that the previously published work could not – that an ICOS-response mechanism has a role in the genetic response to a natural *Salmonella enterica* serovar *Enteritidis* (SE) infection using the chicken model.

Similarly, another EST with higher expression in chickens with low SE burden (resistant birds) is pat.pk0024.f7.f, which was originally structurally-annotated to P0498A12.26, a protein coding region from the plant *Oryza sativa*. Not only is this NCBI record now obsolete, but our re-annotation corrected the structural annotation to chicken LOC693257 NK-lysin. NK-lysin is a known anti-microbial peptide, expressed in T and NK cells [52], that inhibits LPS activity through lipid A binding from several gram-negative bacteria, including *Salmonell*a species [53] [54]. Infection of a porcine model with *Salmonella enterica* serovar *Typhimurium* resulted in higher NK-lysin mRNA expression [54]. Based on our re-annotation, we demonstrate that a mechanism involving the genetic regulation of NK-lysin contributes to a genetic resistance to SE in chicken.

In summary, although bio- and computational-technologies are greatly accelerating functional genomics research, we propose that re-annotation should be the standard first step when analysing functional genomics data. This step is especially valuable for those species in which data and resources are rapidly expanding, including those for which genomic sequence information is only recently available.

## Methods

### EST retrieval

We used the FHCRC Chicken 13K cDNA v.2.0 microarray [19] and the significant differentially expressed mRNAs identified by Zhou et al. [24] as our datasets. The microarray is accessible in the National Center for Biotechnology Information Gene Expression Omnibus (NCBI GEO; http://www.ncbi.nlm.nih.gov/geo/, accession GPL 1836). The table of 15,769 rows was downloaded and filtered for duplicate EST entries, which resulted in 15,227 usable ESTs as described on the GEO website. We used the EST clone IDs for further analysis. The identifiers of the differentially expressed mRNAs were retrieved from the published manuscript by Zhou et al. [24].

### Structural and functional re-annotation

We re-annotated the entire microarray to the most recent structural annotation using the *ArrayIDer* tool [32]. *ArrayIDer* retrieves gene and protein information from both the NCBI UniGene (http://www.ncbi.nlm.nih.gov/./unigene/) and International Protein Index http://www.ebi.ac.uk/IPI/IPIhelp.html). ESTs without structural annotations were searched against the EBI InterPro database [55] using a modified version of InterProScan to allow protein database searches with EST nucleotide sequences translated to amino acid sequences [55]. Functional annotations for the re-annotated structural elements were retrieved from *AgBase* [29], the Gene Ontology Annotation (GOA) chicken database at EBI [56], and manual literature curation. We used the GOA chicken database build 17, published on January 22^nd^ 2007, as a functional annotation baseline available at the time the FHCRC 13K chicken cDNA v2.0 microarray was published. We used the GOA chicken database build 46, published on July 30^th^ 2009, as resource for re-annotations. To compare the original and re-annotated data, we used the 13K microarray and the differentially expressed mRNA data, and both Gene Ontology (GO) and network-based modeling.

### GO modeling

We used *GOSlimViewer* [29] from *AgBase* to group the GO annotations to higher order terms based on the ‘GOA and whole proteome GOSlim’ set for comparing the distribution of major biological groups represented in each dataset. In addition, we used the Gene Ontology Annotation Quality (GAQ) [33] score as a quantitative measure to compare the quality of the assigned functional annotations of the original and re-annotated structural annotations. The GAQ score is a quantitative measure of the functional annotation quality. The GAQ score correlates the number of GO terms (‘breadth’), the GO DAG depth of the annotations, and the type of evidence with which the GO annotation is assigned. The evidence code ranking is designed to give the highest score to GO annotation assigned by direct experimental results. GO annotations assigned automatically receive a lower evidence score in the GAQ score calculation compared to annotations assigned with experimental evidence. In addition to the standard GAQ calculations, we calculated the GO evidence score separately excluding the GO annotations assigned with evidence code “Inferred from Electronic Annotation” (IEA). This allowed us to measure the re-annotation impact on GO annotations assigned based on non-computational assessments.

### Pathway and molecular network analysis

We used the Ingenuity Pathway Analysis application (IPA; Ingenuity® Systems, www.ingenuity.com) to identify and visualize significant canonical pathways represented on the whole microarray and the differentially expressed mRNA datasets of the experiment of Zhou et al. IPA uses publicly available databases and ‘literature curated’ gene information to calculate statistically significant canonical pathways. IPA uses a Fisher's exact test to calculate a P-value determining the probability of the gene associations in the datasets and pathways. To calculate association and gene significance, we used the Agilent 44K chicken microarray (NCBI GEO accession: GPL4993) provided by IPA as a reference list, since this reference is the closest chicken microarray to the FHCRC microarray available in IPA. We used p≤0.05 to select pathways with significant gene representation. We compared the original and re-annotated data based on the represented significant canonical pathways and pathway coverage.

## References

[1] Braga-Neto UM, Marques ET (2006). From functional genomics to functional immunomics: new challenges, old problems, big rewards.. PLoS Comput Biol.

[2] Schena M, Heller RA, Theriault TP, Konrad K, Lachenmeier E (1998). Microarrays: biotechnology's discovery platform for functional genomics.. Trends Biotechnol.

[3] Sellheyer K, Belbin TJ (2004). DNA microarrays: from structural genomics to functional genomics. The applications of gene chips in dermatology and dermatopathology.. J Am Acad Dermatol.

[4] Ashburner M, Ball CA, Blake JA, Botstein D, Butler H (2000). Gene ontology: tool for the unification of biology. The Gene Ontology Consortium.. Nat Genet.

[5] Edgar R, Domrachev M, Lash AE (2002). Gene Expression Omnibus: NCBI gene expression and hybridization array data repository.. Nucleic Acids Res.

[6] Apweiler R, Bairoch A, Wu CH, Barker WC, Boeckmann B (2004). UniProt: the Universal Protein knowledgebase.. Nucleic Acids Res.

[7] Benson DA, Karsch-Mizrachi I, Lipman DJ, Ostell J, Wheeler DL (2004). GenBank: update.. Nucleic Acids Res.

[8] Wu CH, Apweiler R, Bairoch A, Natale DA, Barker WC (2006). The Universal Protein Resource (UniProt): an expanding universe of protein information.. Nucleic Acids Res.

[9] Wortman JR, Gilsenan JM, Joardar V, Deegan J, Clutterbuck J (2009). The 2008 update of the Aspergillus nidulans genome annotation: a community effort.. Fungal Genet Biol.

[10] Salzberg SL (2007). Genome re-annotation: a wiki solution?. Genome Biol.

[11] Ouzounis CA, Karp PD (2002). The past, present and future of genome-wide re-annotation.. Genome Biol.

[12] Gundogdu O, Bentley SD, Holden MT, Parkhill J, Dorrell N (2007). Re-annotation and re-analysis of the Campylobacter jejuni NCTC11168 genome sequence.. BMC Genomics.

[13] Barrett T, Edgar R (2008). Reannotation of array probes at NCBI's GEO database.. Nat Methods.

[14] Chen LL, Ma BG, Gao N (2008). Reannotation of hypothetical ORFs in plant pathogen Erwinia carotovora subsp. atroseptica SCRI1043.. FEBS J.

[15] Daraselia N, Dernovoy D, Tian Y, Borodovsky M, Tatusov R (2003). Reannotation of Shewanella oneidensis genome.. OMICS.

[16] Washietl S, Eisenhaber F (2003). Reannotation of the CELO genome characterizes a set of previously unassigned open reading frames and points to novel modes of host interaction in avian adenoviruses.. BMC Bioinformatics.

[17] Camus JC, Pryor MJ, Medigue C, Cole ST (2002). Re-annotation of the genome sequence of Mycobacterium tuberculosis H37Rv.. Microbiology.

[18] Wood V, Rutherford KM, Ivens A, Rajandream MA, Barrell B (2001). A re-annotation of the Saccharomyces cerevisiae genome.. Comp Funct Genomics.

[19] Burnside J, Neiman P, Tang J, Basom R, Talbot R (2005). Development of a cDNA array for chicken gene expression analysis.. BMC Genomics.

[20] Gupta N, Delrow J, Drawid A, Sengupta AM, Fan G (2008). Repression of B-cell linker (BLNK) and B-cell adaptor for phosphoinositide 3-kinase (BCAP) is important for lymphocyte transformation by rel proteins.. Cancer Res.

[21] Neiman PE, Kimmel R, Icreverzi A, Elsaesser K, Bowers SJ (2006). Genomic instability during Myc-induced lymphomagenesis in the bursa of Fabricius.. Oncogene.

[22] Rocques N, Abou Zeid N, Sii-Felice K, Lecoin L, Felder-Schmittbuhl MP (2007). GSK-3-mediated phosphorylation enhances Maf-transforming activity.. Mol Cell.

[23] Wang X, Rosa AJ, Oliverira HN, Rosa GJ, Guo X (2006). Transcriptome of local innate and adaptive immunity during early phase of infectious bronchitis viral infection.. Viral Immunol.

[24] Zhou H, Lamont SJ (2007). Global gene expression profile after Salmonella enterica Serovar enteritidis challenge in two F8 advanced intercross chicken lines.. Cytogenet Genome Res.

[25] Smith C, McFarland DC, Oliverira HN, Rosa GJM, Sanborn AM

[26] Consortium ICGS (2004). Sequence and comparative analysis of the chicken genome provide unique perspectives on vertebrate evolution.. Nature.

[27] Buza TJ, McCarthy FM, Burgess SC (2007). Experimental-confirmation and functional-annotation of predicted proteins in the chicken genome.. BMC Genomics.

[28] Eyras E, Reymond A, Castelo R, Bye JM, Camara F (2005). Gene finding in the chicken genome.. BMC Bioinformatics.

[29] McCarthy FM, Bridges SM, Wang N, Magee GB, Williams WP (2007). AgBase: a unified resource for functional analysis in agriculture.. Nucleic Acids Res.

[30] Neerincx PB, Rauwerda H, Nie H, Groenen MA, Breit TM (2009). OligoRAP - an Oligo Re-Annotation Pipeline to improve annotation and estimate target specificity.. BMC Proc.

[31] Richardson MK, Crooijmans RP, Groenen MA (2007). Sequencing and genomic annotation of the chicken (Gallus gallus) Hox clusters, and mapping of evolutionarily conserved regions.. Cytogenet Genome Res.

[32] van den Berg BH, Konieczka JH, McCarthy FM, Burgess SC (2009). ArrayIDer: automated structural re-annotation pipeline for DNA microarrays.. BMC Bioinformatics.

[33] Buza TJ, McCarthy FM, Wang N, Bridges SM, Burgess SC (2008). Gene Ontology annotation quality analysis in model eukaryotes.. Nucleic Acids Res.

[34] Pruess M, Fleischmann W, Kanapin A, Karavidopoulou Y, Kersey P (2003). The Proteome Analysis database: a tool for the in silico analysis of whole proteomes.. Nucleic Acids Res.

[35] Brenner SE (1999). Errors in genome annotation.. Trends Genet.

[36] Devos D, Valencia A (2001). Intrinsic errors in genome annotation.. Trends Genet.

[37] Jones CE, Brown AL, Baumann U (2007). Estimating the annotation error rate of curated GO database sequence annotations.. BMC Bioinformatics.

[38] Schnoes AM, Brown SD, Dodevski I, Babbitt PC (2009). Annotation error in public databases: misannotation of molecular function in enzyme superfamilies.. PLoS Comput Biol.

[39] Kim KM, Sung S, Caetano-Anolles G, Han JY, Kim H (2008). An approach of orthology detection from homologous sequences under minimum evolution.. Nucleic Acids Res.

[40] Moreno-Hagelsieb G, Latimer K (2008). Choosing BLAST options for better detection of orthologs as reciprocal best hits.. Bioinformatics.

[41] Wall DP, Deluca T (2007). Ortholog detection using the reciprocal smallest distance algorithm.. Methods Mol Biol.

[42] European Molecular Biology Laboratory (EMBL) - European Bioinformatics Institute (EBI) - Genome Annotation Scores (GAS) - http://www.ebi.ac.uk/integr8/HelpAction.do?action=searchById&refId=60

[43] Kemmer D, Podowski RM, Yusuf D, Brumm J, Cheung W (2008). Gene characterization index: assessing the depth of gene annotation.. PLoS One.

[44] Harel A, Inger A, Stelzer G, Strichman-Almashanu L, Dalah I (2009). GIFtS: annotation landscape analysis with GeneCards.. BMC Bioinformatics.

[45] Tian L, Greenberg SA, Kong SW, Altschuler J, Kohane IS (2005). Discovering statistically significant pathways in expression profiling studies.. Proc Natl Acad Sci U S A.

[46] Rudd CE, Taylor A, Schneider H (2009). CD28 and CTLA-4 coreceptor expression and signal transduction.. Immunol Rev.

[47] Shilling RA, Bandukwala HS, Sperling AI (2006). Regulation of T:B cell interactions by the inducible costimulator molecule: does ICOS “induce” disease?. Clin Immunol.

[48] van Berkel ME, Oosterwegel MA (2006). CD28 and ICOS: similar or separate costimulators of T cells?. Immunol Lett.

[49] Hutloff A, Dittrich AM, Beier KC, Eljaschewitsch B, Kraft R (1999). ICOS is an inducible T-cell co-stimulator structurally and functionally related to CD28.. Nature.

[50] de Jong YP, Rietdijk ST, Faubion WA, Abadia-Molina AC, Clarke K (2004). Blocking inducible co-stimulator in the absence of CD28 impairs Th1 and CD25+ regulatory T cells in murine colitis.. Int Immunol.

[51] Vidric M, Bladt AT, Dianzani U, Watts TH (2006). Role for inducible costimulator in control of Salmonella enterica serovar Typhimurium infection in mice.. Infect Immun.

[52] Andersson M, Gunne H, Agerberth B, Boman A, Bergman T (1995). NK-lysin, a novel effector peptide of cytotoxic T and NK cells. Structure and cDNA cloning of the porcine form, induction by interleukin 2, antibacterial and antitumour activity.. EMBO J.

[53] Andersson M, Girard R, Cazenave P (1999). Interaction of NK lysin, a peptide produced by cytolytic lymphocytes, with endotoxin.. Infect Immun.

[54] Meurens F, Berri M, Auray G, Melo S, Levast B (2009). Early immune response following Salmonella enterica subspecies enterica serovar Typhimurium infection in porcine jejunal gut loops.. Vet Res.

[55] Quevillon E, Silventoinen V, Pillai S, Harte N, Mulder N (2005). InterProScan: protein domains identifier.. Nucleic Acids Res.

[56] Barrell D, Dimmer E, Huntley RP, Binns D, O'Donovan C (2009). The GOA database in 2009–an integrated Gene Ontology Annotation resource.. Nucleic Acids Res.

